# Impact of supervised exercise training on pulmonary function parameters, exercise capacity and Irisin Biomarker in Interstitial lung disease patients

**DOI:** 10.12669/pjms.36.5.1795

**Published:** 2020

**Authors:** Rahma Mohammad Alyami, Abdulrahman Mohammed Alhowikan, Abdullah Rashed Alharbi, Ghada AL-Nafisah

**Affiliations:** 1 Rahma Mohammad Alyami, MS. College of Medicine, King Khalid University, Abha, Saudi Arabia; 2 Abdulrahman Mohammed Alhowikan, PhD. Department of Physiology, Faculty of Medicine, King Saud University, Riyadh, Saudi Arabia; 3 Abdullah Rashed Alharbi, MBBS. Department of Medicine, King Saud University, Riyadh, Saudi Arabia; 4 Ghada AL-Nafisah, MS. Research Center, College of Medicine, King Saud University, Riyadh, Saudi Arabia

**Keywords:** Supervised exercise training, Irisin, Interstitial Lung Disease

## Abstract

**Objectives:**

To assess the impact of supervised exercise training (SET) on pulmonary function Parameters, exercise capacity and Irisin biomarker in Interstitial Lung Disease (ILD) patients.

**Methods:**

Ten (10)patients with ILD and 18 healthy controls of age between 30-40+ years were selected for 8-week SET program. Before and after SET all subjects performed exercise capacity six minutes’ walk test (6MWT), heart rate (HR) changes were recorded, shortness of Breath Respiratory Questionnaire (SOBQ) was obtained and Irisin levels were measured by Enzyme-Linked Immunosorbent Assay (ELISA). This interventional study was carried out at Department of Physiology, Faculty of Medicine, King Khalid University Hospital, King Saud University, Riyadh, Saudi Arabia, from October 2018 to February 2019.

**Results:**

Mean six minutes’ walk distance (6MWD) was 395 ± 68.4 m at 1st visit increased significantly (p=0.001) to 458.8± 87.1 mat 15 visit. However, 6MWD values found significantly higher in controls (517.4±84.1 m; 561.7±81.6 m; p=0.013) than ILD patients. Overall change (difference between post and pre exercise) in HRvalue was recorded lower in ILD patients (30-35 bpm) as compared to controls (40-45 bpm). Moreover, ILD patients had overall higher SOBQ score than controls. Pre SET Irisin levels of ILD patients (4.24 ±1.73 pg/ml) and controls (3.43 ±1.04pg/ml) were found unchanged dafter SET (4.48±2.02pg/ml, 3.39 ±1.41pg/ml, p=0.677, p=0.093)respectively. However, patients Irisin values were found higher as compared to controls before and after SET.

**Conclusion:**

Exercise capacity and Dyspneain patients with ILD were improved after 8-week of SET program. No major changes in Irisin levels among patients with ILD and controls were observed. Additional research requires to be carried out on large number of subjects to deter Minutese the advantages of exercise in ILD.

## INTRODUCTION

In both healthy subjects and patients with chronic diseases, physical fitness and regular exercise have been shown to improve functional health and reduce mortality risk.^1^Exercise training (ET) and rehabilitation programs in patients with chronic respiratory disease have been reported to improve signs and exercise ability, but the degree and period of the outcome in precise situations need to be completelyexplained.^2^Exercise training is an essential factor of evidence-based controlling programs for several long-lasting cardiac and respiratory disorders. There is a substantial indication that exercise activity encourages major improvement in exercise tolerance and useful rehabilitation for patients with interstitial lung disease (ILD).^3^

The Interstitial Lung Disease isa chronic lung condition categorized by dyspnea on exertion and deprived health related quality of life.^4^The role of exercise training and pulmonary rehabilitation programmes in the ILD patients is still uncertain. A number of exercise training studies recommend that pulmonary rehabilitation programs that contain exercise training may recover functional capacity and quality of life in the ILD^5^, however, there is noticeable difference in results between individuals.^6^Physical exercise offers potential as a useful treatment for subjects with ILD, with advancements in six-minute’s walk distance (6MWD), dyspnoea, and exercisecapacity.^7^Recently Bonini et al^8^reviewedandexplained the respiratory physiology of ILD, together at rest and throughout exercise. However according to clinical practice guidelines the quality of the data supporting the use of exercise training^9^islimited and that the longstanding benefits are not clear.^10^

In sports biomarkers are main factors to evaluate the effect of exercise on altered systems, tissues and organs. Moreover, blood biomarkers can be used as tool to assess the effect of exercise training on patients with ILD. In response to physical exercise, muscle tissue secrete myokine called Irisin, and it is believed that these myokines are involved in physical activity induced positive healthresults.^11^However in humans, the regulation of Irisin by exercise is not clear. Huh etal^12^ examined the effect of acute and chronic whole-body vibration exercise on circulating irisin levels in young healthy subjects. In previous studies Irisin has been recommended to mediate the useful effects of exercise. Thus, Irisin could be a balanced biomarker for diseases linked with physical inactivity.^13^However, many studies have reported increase in Irisin blood concentration after acute exercise training.^14^A number of reports have established the improved circulating Irisin in response to acute bouts of exercise, mostly using treadmill or bicycleexercise.^15^ It remains to be elucidated whether other types of exercise can increase irisin acutely or after a period of training. Moreover, whether main physiological ordemographic variables play a part in estimation of irisin’s response to exercise remains to be clarified.

The main object of this study was to assess the impact of supervised exercise training (SET) on pulmonary function Parameters, exercise capacity and Irisin biomarker in ILD patients.

## METHODS

Interstitial Lung Disease (ILD) patients (n=10, male=3; female 7)with stable condition and healthy controls (n=18, male=7; female=11) aged between 30-40+ years were recruited in this interventional design (single-arm clinical trial (SACT) study, conducted at Department of Physiology, Faculty of Medicine, King Khalid University Hospital (KKUH), King Saud University, Riyadh, Saudi Arabia, from October 2018 to February 2019.

### Inclusion criteria

The inclusion criteria for SET program in ILD has been broad and participants have had a wide range of ILD diagnoses (Lung fibrosis, Rhumatoid arthtitis, Sclerodema, Dermatomyositis, Ankylosing spondylitis). The patients with stable condition, nonsmokers, having dyspnea on exertion, on medical treatment and not involved in any exercise program in the last 12 months were included. All subjects participated in this study were free from any serious risk of exercise training according to the guidelines of ACC/AHA for exercise testing, American College of Sports Medicine Guidelines for Exercise Testing and Prescription.^16^Interstitial Lung Disease patients were referred from pulmonary clinic at King Khalid University Hospital (KKUH) and Controls were recruited from the staff at the King Saud University. The control subjects were medically free, nonsmokers and their pulmonary function tests were normal.

### Exclusion criteria

ILD subjects with long term Oxygen (O_2_) supply, predominant respiratory disease other than ILD, unstable cardiac disease (such as active Ischemic Heart disease, arrhythmia, and pacemaker). Also, subjects who had a musculoskeletal disability (e.g., active arthritis), any comorbidities that prevent exercise, a history of syncope on exertion, communication or transport difficulties and history of arterial SaO_2_ during exercise (equal or less than 90 % on peak exercise), were all excluded from the study.

Participant having oxygen saturation (SaO_2_) below 90%, required O_2_ supply. All exercise protocol was supervised and performed with oxygen supplementation 2 L/Minutes via intranasal cannula to avoid unwanted shortness of breath (SOB). Full written consent obtained from the participants prior to the study. Adequate level of confidentiality and privacy of subjects was established. Study protocol was approved by the College of Medicine King Saud University Institutional Review Board (Ref. No. 17/0591/IRB, dated Oct. 5, 2017).

Pulmonary function test(PFT) was carried out by previously recommended techniques.^17^Measurements of participant’s body mass composition, blood pressure, oxygen desaturation SpO2, were recorded before and after the exercise. The endurance exercise training 2 days per week for 8 weeks were carried out by using cycle ergometer and arm-ergometer exercise as described previously.^16^, All outcome measures were determined at baseline (visit 1) and after completion of eight weeks of the exercise intervention (visit 15). Following variable indicators were measured before and after SET.

### Shortness of breath questionnaire (SOBQ)

Both patients and healthy subjects were asked to fill SOBQ. ) It is a multi-dimensional instrument to measure the three factors of dyspnea (intensity, quality and emotional responses to this sensation). It is a valid and reliable tool used to indicate severity of shortness of breath previously^18^. The ***(SOBQ)*** involves twelve descriptor items on a scale of none (0), mild (1), moderate (2), or severe (3). Total scores from the ***(SOBQ)*** range from 0 to 36, with higher scores showing greater severity.

### Six Minutesutes Walk Test (6MWT)

The 6MWTwas used^19^for estimating exercise capacity in both ILD patients and controls. The reproducibility of the 6MWD has been confirmed in ILD^19^. Participants were trained to walk from one end to the other of long uncrowded 50 m long hallway at their own speed, while trying to cover as much as possible distance in 6 minutes.. Finally covering distance was recorded at the end of six-minute’s waking before and after SET program.

### Heart Rate (HR)

Heart rate is the speed of the heartbeat measured by the number of contractions (beats) of the heart per minute (bpm). The heart rate can vary according to the body’s physical needs, including the need to absorb oxygen and excrete carbon dioxide. Heart rate (HR) of all participants was recorded before and after SET sessions.

### Blood Sample Collection

Blood samples in 5ml EDITA tube (plasma) and 5 ml of plain tube (serum) were collected from patients and healthy controls before and after SET session. Collected blood was stored at -80 °C and later used to measure irisin levels by ELISA.

### Statistical Analysis

Data were analysed by using the statistical software package SPSS, version 21 and werepresented as mean ±SD. The difference in study variables(6MWT, HR, SOBQ and Irisine levels)in both ILD and control groups were calculated before and after SET. The within and between-group differences were analysed using repeated measures analysis of variance. Between groups comparisons of baseline characteristics were carried out using an unpaired t-tesafter checking for normal distribution. The level of significancewas set at p≤0.05. Irisin Protein values were not normally distributed. Mann-Whitney Test was used.

## RESULTS

The impact of eight (8) week SET program on 6MWT, SOBQ HRandIrisine levels were measured before and after each exercise session. Participants were from different age group and sample below and above 40 years shows significant difference (p=0.008) however body fat percent values, as quantified by body-mass index (BMI) (kg/m 2) were found non-significant (p=0.228).

Six Minutesutes’ walk test (6MWT) results of the sampled study groups are shown in Table-I. All patients with ILDand controls improved 6MWD score after SET program. Mean distance of 6MWT in ILD patientsand controls increased significantly at 15 visit. However, 6MWT scorefound significantly higher in controls than ILD patients.

**Table-I:** Six Minutesutes’ walk test (6MWT) of the sampled study groups.

6MWTdistance (meter)		Group		p^1^

		Patient (n=10)	Control (n=18)	
Visit 1	Range	300-550	392-656	0.001*
	Mean (SD)	395 (68.4)	517.4 (84.1)	
Visit 15	Range	350-600	420-670	0.01*
	Mean (SD)	458.8 (87.1)	561.7 (81.6)
p^2^		0.001*	0.001	

p^1^: adjusted p value of independent t-testbetween control and patient group.

p^2^: adjusted p value of paired t-testbetween same group.

* p < 0.05 (significant)

**Fig.1:**
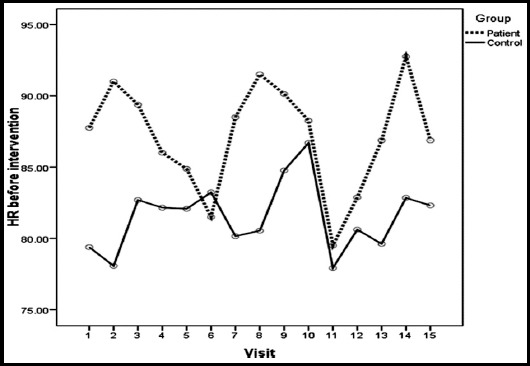
Trend of HR(difference between pre and post SET) before intervention at each visit among the study groups.

**Fig.2:**
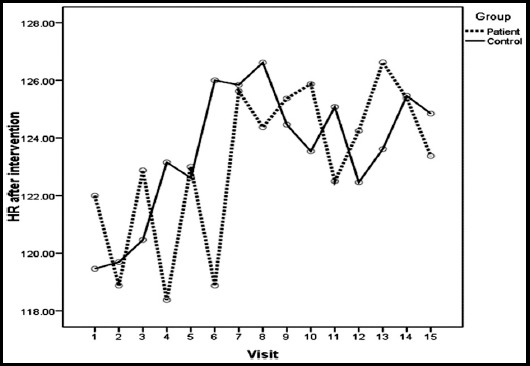
Trend of HR (difference between pre and post SET) after intervention at each visit among the study groups.

Trends in HR beforeand afterSETrespectively at each visit among the study groups are shown in Fig-1 and II. Patients group comparatively has higher HR than controls before starting SET (Fig.1) and after completing SET (Fig.2). Table-II shows overall change (difference between pre and post SET) in heart rate (HR) ofILD patents (30-35 bpm) compared tocontrols(40-45 bpm)at each visit.

**Table-II:** Overall Change (difference between pre and post SET) in Heart rate (HR) in the study groups.

Visit	HR (bpm) of ILD patients (n=10)			HR ((bpm) of Controls (n=18)			p

	Pre SET mean (SD)	Post SET mean (SD)	Change in HR mean (SD)	Pre SET mean (SD)	Post SET mean (SD)	Change in HR mean (SD)	
1	85.1 (10.1)	117.4 (14.7)	32.30 (13.22)	81.9 (10.1)	124.2 (13.1)	42.28 (11.79)	0.050*
2	88.6 (7.6)	115.5 (10.6)	26.90 (9.05)	80.1 (10.3)	122.4 (10.3)	42.33 (9.85)	0.001*
3	90.3 (13.6)	121.9 (8.4)	31.56 (18.84)	84.2 (9.8)	123.3 (12.8)	39.17 (14.67)	0.258
4	85.8 (9.4)	117.4 (7.7)	31.67 (12.61)	83.3 (10.9)	125.4 (12.0)	42.06 (11.58)	0.045*
5	84.3 (9.7)	122.4 (5.2)	38.11(8.75)	82.8 (11.5)	125.4 (13.4)	42.63 (13.71)	0.384
6	72.4 (28.4)	118.9 (6.8)	37.38 (11.26)	83.4 (7.3)	127.0 (12.0)	43.64 (11.42)	0.228
7	88.5 (11.6)	125.6 (6.3)	37.13 (13.16)	80.2 (7.0)	125.8 (9.9)	45.69 (14.02)	0.180
8	91.5 (5.5)	124.4 (8.4)	32.88 (11.53)	80.5 (10.1)	126.6 (9.5)	46.08 (12.53)	0.026*
9	90.1 (8.5)	125.4 (9.5	35.25 (8.24)	84.8 (8.1)	124.5 (6.9)	39.69 (5.11)	0.141
10	88.3 (9.6)	125.9 (9.0)	37.63 (9.15)	86.7 (8.9)	123.5 (12.2)	36.85 (12.14)	0.878
11	79.5 (9.6)	122.5 (7.2)	43.00 (11,74)	77.9 (7.5)	125.1 (10.3)	47.15 (9.52)	0.385
12	82.9 (6.6)	124.3 (9.4)	41.38 (12.98)	80.6 (5.5)	122.5 (8.1)	41.85 (11.58)	0.932
13	86.9 (9.5)	126.6 (8.7)	39.75 (8.29)	79.6 (7.8)	123.6 (11.6)	44.00 (12.88)	0.417
14	92.8 (4.9)	125.4 (5.8)	32.63 (5.78)	82.8 (6.8)	125.5 (6.9)	42.62 (7.42)	0.004*
15	86.9 (8.7)	123.38 (6.4)	36.50 (11.25)	82.3 (7.3)	124.85 (9.14)	42.54 (8.08)	0.168

* p < 0.05 (significant).

Patients with ILD had higher Score of overall SOBQ than controls and the score reduced significantly (p=0.012) after SET, however overall SOBQ score of control group did not changed remarkably (p=0.239).

Table.III shows Irisin levelsofILD patientswhich did not changed significantly after SET. Furthermore, there was no significant difference in pre-exercise Irisin levels and post exercise Irisin levels of patient and control groups was observed. However, these values mayhave consideredclinically significant.

**Table-III:** Change in Irisine levels before and after SET among the studygroups.

Factor		Group		p^1^

		Patient	Control	
IRISIN Before	Range	1.91 - 6.78	1.09 - 4.80	0.22
	Mean (SD)	4.24 (1.73)	3.43 (1.04)	
IRISIN After	Range	2.45 - 8.74	1.35 - 5.71	0.18
	Mean (SD)	4.48 (2.02)	3.39 (1.41)
p^2^		0.68	0.09	

p^1^: adjusted p value of independent t-testbetween control and patient group.

p^2^: adjusted p value of paired t-testbetween same groups.

* p < 0.05 (significant).

## DISCUSSION

This is the first interventional design (single-arm clinical trial) studyof SET in ILD patients in Kingdom of Saudi Arabia. In this study all patients (male=3, female=7) with IL improved 6MWT significantly which is consistent with earlier reports, showed a significant improvement in 6MWD following pulmonary rehabilitation in ILD patients.^6^Our results showed the overall mean improvement of 53 m in 6MWD which was greater than the minimal important difference(MID) (30–33 m)^9^and previously reported, (35 m) for subjects with ILD.^5^Though increases in 6MWD beyond the MCID^6^have been described after exercise training (ET) in patients with ILD, however, the mechanism by which this increase wasarbitrated was not known. Moreover, our results suggested that clinically relevant improvement in symptoms did occur in most participants following exercise training.6-MWT distance is a possibly useful biomarker of chronic obstructive pulmonary disease (COPD) severity, which is influenced by muscle weakness, pulmonary vascular disease.^20^Thedetected improvement in 6-MWDwasassumed to be clinically significant. Moreover, causes or severity of ILD did not predict temporary improvement in 6MWD or signs. This recommends that exercise training may be effective throughout the entire scale of disease in succeeding short-term benefits. Therefore, all patients with ILD should be provided with the chance to carry out exercise training, though timely referral is suggested to support longer-permanent effects. Other approaches such as a longer-term intervention, maintenance exercise programmes or regular sessions of exercise training may also support longer-lasting benefits but further research is required in this main region.

In spite of the limited improvement in those with ILD, some symptomatic benefit was attained with variations in SOBQ and HR symptoms beyond the MCID as 53. Generally, the SOBQ and HR score improved after exercise. Further investigation is necessary to explain the usefulness of the SOBQ in determining change in dyspnoea in ILD. These facts have important clinical significance and practical associations, since proven and effective therapies for ILD are restricted.

“Over 200 etiologies can result in ILD which is identified by irreversible fibrotic reorganization of the lung parenchyma. Interstitial fibrosis creates a barrier to gas exchange at the alveolar-capillary interface, and decreases lung dispensability. “Consequently, hemoglobin oxygenation is reduced and the work of ventilation is increased, leading to reduced cardiorespiratory function.^21^ In addition to low cardiorespiratory capacity, skeletal muscle weakness has also been linked with poor 6MWT performance in patients with ILD, which is the most often used method for assessing exercise capacity in these ILD patients.^22^

The impact of SET on development of physiological and clinical results in ILD can be described by numerous mechanisms. ILD patients generally show compromised lung function and inefficient breathing patterns as part of the restrictive pathophysiology.^23^It is likely that repetitive stimulus of high ventilatory stresses throughout exercise periods, chest expansion during deep breathing exercises and stretching of the thoracic muscles that were used in our program resulted in a more efficient breathing pattern, recovered strength of respiratory muscles, improved pleural elasticity and pulmonary compliance within the lung tissue, and decreased dyspnea perception following the SET program.^23^SET program duration has been under debit. The perfect duration of pulmonary rehabilitation (PR) for people with ILD is uncertain. The British guidelines for PR suggested programs of 6–12 weeks ‘length, but no recommendations particular to ILD were made.^25^

Our results showed no significant difference between the concentration of pre-exercise Irisin level and post exercise level in patient as well as in control groups. However, patients Irisin values were higher than control group which may considered clinically significant.

Several studies have examined the effect of exercise on Irisin concentration and have reported contradictory results.^25^Various studies using different physical exercise methods have also failed to identify the link between Irisin levels and exercise.^25^ However, various studies have shown an increase in circulating levels of Irisin after exercise training.^26^

In humans, the period of exercise appears to be important for variations in circulating levels of Irisin^12^The type of acute exercise might affect Irisin, some studies suggested that aerobic exercise, as well as other resistance exercises or heavy strength training, stimulates the increase in circulating levels of Irisin.^26^Severalessential questions remain to be answered. As in vitro muscle contraction does not stimulate the release of Irisin, the high serum levels of the hormone that are observed after acute exercise might be the result of muscle damage or of unidentified biochemical and molecular changes.^27^

It was assumed^28^ that Irisin should respond to several acute and chronic exercise protocols. On the other hand, whether an exercise induced effect on Irisin parameteroccurs or not persists a rather debatable subject, since experimental data differ between studies giving rise to a discussion that emphases on

1. Whether Irisin is essential in human metabolism.
2. Whether exercise is an effective incentive for Irisin release.
3. What are the physiological effects of irisin in human exercise?
4. Whether exercise encourages FNDC5 expression in different organs.
5. The suitability of the existing techniques to measure circulating irisin in human samples.

In spite of strong indication for the advantages of SET in ILD, this area remains to develop. Essential concerns still remain to be answered including the best design and content for SET; techniques to cover period of advantage; and how SET can best be customized to the multifaceted requirements of subjects with ILD. For example, the best approaches to offer complete SET for subjects with advanced or last-stage disease have not been investigated. Additional work required to describe the non-exercise factors of SET that provide to best results, including psychosocial support, education, behavioral intervention and group therapy.

### Limitations of the study

The small sample size and heterogeneous sample group with respect to inclusion of patients with numerous ILD etiologies prevent the capability to generalize our results. Due to the importance of promoting physical health in ILD patients, further investigations in large randomized controlled studies are required with different training modalities to optimize the SET programmes for ILD.

In summary, current studies recommended that SET is effective through the spectrum of ILD and could be offered to all patients who are symptomatic on exertion, irrespective of underlying diagnosis. Although the clinical effectiveness of SET in ILD is increasingly understood, clarification of cost effectiveness of SET would offer motivation for commissioners and policy makers to confirm this intervention is generally available.^29^The benefit of exercise training could differ according to disease severity and aetiologyand the tiMinutesg of exercise training may matter for particular types of ILD. Due to the importance of promoting physical health in ILD patients, further investigation in large randomized controlled studies should address different training modalities to optimize the exercise training programs for ILD.

## CONCLUSION

This study demonstrates that the SET program is safe and effective for patients with ILD, delivering clinically important improvements in dyspnea and exercise capacity. This strengthens the recommendation for the role of SET as a part of a standard comprehensive treatment for ILD patients. Our results showed that SET does not affect circulating Irisin levels in ILD patients. However, further study is needed on large scale to assess the physiological impact of the Irisin in in ILD patients and also confirm or refute a role of Irisin in exercise-induced energy metabolism.

### Authors’ Contribution

**RMA:** Did the data analysis and data interpretation.

**AMA:**Responsible and accountable for the accuracy or integrity of the work.

Conceived the idea and designed the study. Approval of the final version to be published.

**ARA:**Drafting and revising the paper.

**GAN:** Literature search and final review.
